# Characterization of the Rabbit Neonatal Fc Receptor (FcRn) and Analyzing the Immunophenotype of the Transgenic Rabbits That Overexpresses FcRn

**DOI:** 10.1371/journal.pone.0028869

**Published:** 2012-01-11

**Authors:** Ana Paula Catunda Lemos, Judit Cervenak, Balázs Bender, Orsolya Ivett Hoffmann, Mária Baranyi, Andrea Kerekes, Anita Farkas, Zsuzsanna Bősze, László Hiripi, Imre Kacskovics

**Affiliations:** 1 Agricultural Biotechnology Center, Gödöllő, Hungary; 2 ImmunoGenes Kft, Budakeszi, Hungary; 3 Department of Immunology, Eötvös Loránd University, Budapest, Hungary; INRA, France

## Abstract

The neonatal Fc receptor (FcRn) regulates IgG and albumin homeostasis, mediates maternal IgG transport, takes an active role in phagocytosis, and delivers antigen for presentation. We have previously shown that overexpression of FcRn in transgenic mice significantly improves the humoral immune response. Because rabbits are an important source of polyclonal and monoclonal antibodies, adaptation of our FcRn overexpression technology in this species would bring significant advantages. We cloned the full length cDNA of the rabbit FcRn alpha-chain and found that it is similar to its orthologous analyzed so far. The rabbit FcRn - IgG contact residues are highly conserved, and based on this we predicted pH dependent interaction, which we confirmed by analyzing the pH dependent binding of FcRn to rabbit IgG using yolk sac lysates of rabbit fetuses by Western blot. Using immunohistochemistry, we detected strong FcRn staining in the endodermal cells of the rabbit yolk sac membrane, while the placental trophoblast cells and amnion showed no FcRn staining. Then, using BAC transgenesis we generated transgenic rabbits carrying and overexpressing a 110 kb rabbit genomic fragment encoding the FcRn. These transgenic rabbits – having one extra copy of the FcRn when hemizygous and two extra copies when homozygous - showed improved IgG protection and an augmented humoral immune response when immunized with a variety of different antigens. Our results in these transgenic rabbits demonstrate an increased immune response, similar to what we described in mice, indicating that FcRn overexpression brings significant advantages for the production of polyclonal and monoclonal antibodies.

## Introduction

Maintenance of antibody (Ab) levels requires continuous secretion of immunoglobulin (Ig) by plasma cells and protection from degradation. IgG is a class of Abs that is unique to mammals. It is the most abundant Ab in serum and is also passively transferred to mammalian offspring. From the standpoint of therapeutic, diagnostic or research Ab reagent production, it is the most important Ab class worth serious consideration when preparing an Ab reagent.

In 1958, Brambell described a saturable receptor that mediates the transport of maternal gamma-globulin to the fetus [1]; he then inferred the presence of a similar or identical receptor that protected gamma-globulin from catabolism to make it the longest surviving of all plasma proteins [2]. At about the same time, it was shown that 7S γ-globulin (IgG) is the fraction of Ig that was protected by such a mechanism [3]; a few years later, it was also shown that IgG mediates maternal immune transport in mammals [4], [5].

The neonatal Fc receptor (FcRn) was first identified in the 1970s as the protein that mediates transfer of maternal, milk-borne IgGs across the rodent neonatal intestine [6]. Subsequently, FcRn was shown to be a heterodimer of two polypeptides that binds IgG at the CH2–CH3 interface, in a strictly pH dependent way with binding occurring at slightly acidic pH and no detectable binding at pH 7.4 [7], [8]. It was finally characterized as composed of an MHC class-I like α-chain and beta 2-microglobulin (β2m) [9]. FcRn has proven to be a key player in regulating the transport of IgG within and across cells of diverse origin and it also serves to rescue IgG and albumin from degradation, thereby prolonging their half-lives [10]. IgG protection was originally thought to be mediated by capillary endothelial cells [11]; however, recent findings suggest that this process also occurs in hematopoietic cells [12], [13], or even in mammary epithelial cells during lactation [14]. FcRn orthologous have been isolated from mouse, rat, human, sheep, cow, possum, pig and camel, suggesting that this receptor is present in essentially all mammalian species [10]. More recently, several publications have shown that FcRn plays major roles in antigen-IgG immune-complex phagocytosis by neutrophils [15], and also in antigen presentation of IgG immune complexes by professional antigen presenting cells [16], [17], [18], [19].

The existence of the Brambell receptor was also hypothesized in rabbits when early data showed receptor mediated maternal gamma-globulin transport through rabbit yolk sac [20] where the receptor was localized to the glycocalyx-coated vesicles as well as the glycocalyx-coated brush border [21], and that increased serum gamma-globulin resulted in faster catabolism in this species [22]. It was also shown that the rabbit IgG half-life depends on the Fc fragment [3], and the maternal IgG transport through yolk sac is CH2 domain dependent [23]. Another study indicated that the mouse and rabbit IgGs, pre-incubated with staphylococcal protein A (SpA), had much shorter half-lives [24] and thus suggested that those amino acid residues that are involved in IgG protection or maternal transport locate in the CH2–CH3 domain interface. The overlapping residues at the CH2–CH3 domain interface with the FcRn and SpA binding sites were confirmed a decade later [25]. Despite the fact that rabbit served as an important model in studying maternal immunoglobulin transport and IgG catabolism from the beginning, the FcRn receptor and its function has not been characterized in this species.

We, and others, have shown that higher than normal expression levels of FcRn reduced exogenous IgG catabolism in transgenic mice, resulting in higher circulating levels of IgG [14], [26], [27]. Our more recent studies have demonstrated that FcRn overexpression in transgenic (Tg) mice enhances the expansion and diversity of antigen-specific B cells and plasma cells in secondary lymphoid organs [28]. Furthermore, we found that these Tg mice were able to mount robust humoral response against weakly immunogenic antigens and to improve hybridoma production efficiency without any sign of autoimmunity [29], [30], [31]. Since rabbit is one of the most important sources of polyclonal, and recently also monoclonal, Abs for therapeutic, diagnostic and research applications, adaptation of the FcRn overexpression technology in this species would bring significant advantages. Consequently, we decided to clone and characterize the rabbit FcRn, create Tg rabbits that overexpress this receptor, and analyze their humoral immune response.

## Results

### 1. Characterization of the rabbit FcRn α-chain cDNA

To isolate the full length of the rabbit FcRn α-chain, we first synthesized cDNA from the RNA isolated from rabbit liver and spleen. We then performed 3′-RACE and 5′-RACE, using rabbit specific primers which were designed based on available EST sequence data. The obtained composite DNA sequence of 1415 bp contained a segment of the 5′-untranslated (UT) region, the α1, α2, α3 domains, the transmembrane (TM) domain, the cytoplasmic (CYT) domain and ended with the 3′-UT region of the rabbit cDNA. (The sequence data have been submitted to the NCBI Nucleotide Sequence Databases under the accession number: JN558833). The data were compared to other published FcRn α-chain sequences, and showed a 99% homology (with three mismatches) to a rabbit FcRn α-chain cDNA that was deposited in GenBank (NM_001122937.1) albeit without functional characterization and publication. The rabbit FcRn α-chain specific cDNA sequence we obtained showed high homology to its orthologous analyzed thus far.

### 2. Rabbit FcRn α-chain mRNA is expressed in all analyzed tissues and cells; the rabbit embryos start to express FcRn by 6 dpc, close to implantation time

**Figure 1:**
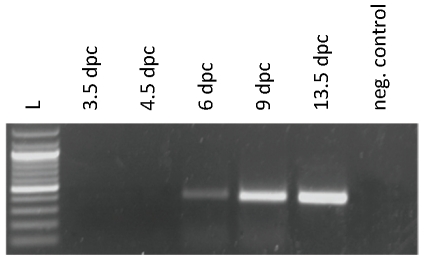
Rabbit embryos start to express FcRn by 6 dpc, close to the implantation time. Rabbit FcRn α-chain expression was analysed in rabbit blastocysts and embryos at different time points by PCR. L-ladder, 1–3.5 dpc rabbit blastocyst, 2–4.5 dpc rabbit blastocyst, 3–6 dpc rabbit embryo, 4–9 dpc rabbit embryo, 5–13.5 dpc rabbit embryo, 6 - negative control (DNA omitted).

Subsequently, we examined the tissue distribution of rabbit FcRn α-chain using PCR. We could detect expected FcRn specific amplicons from all tissues and cells we analyzed including kidney, ovary, uterus, salivary gland, lymph node, lung, brain, liver, spleen, small intestine, placenta, amnion and yolk sac, rabbit blastocyst and embryo as well as peritoneal macrophages (data not shown). We have also investigated the expression of the rabbit FcRn α-chain in rabbit blastocysts and embryos in different time points (3.5; 4.5; 6; 9 and 13.5 dpc) by PCR. We found that rabbit embryos start to express FcRn by 6 dpc, close to the implantation time (**Fig. 1**). There is some similarity in the expression of FcRn in rabbit and mouse because this expression occurs before implantation in both species, but there is also differences because rabbit expresses the gene in the late preimplantation stages only, as mouse expresses it in all preimplantation stages tested [32].

### 3. Characterization of the deduced amino acid sequence of the rabbit FcRn α-chain

The full-length transcript of the rabbit FcRn α-chain we isolated is composed of three extracellular domains (α1-α2-α3), a transmembrane (TM) region and a cytoplasmic (CYT) tail; the molecular weight of the matured rabbit FcRn α-chain protein is estimated to be 38 kDa (without carbohydrate side chain) based on its amino acid sequence. Similarly to the cDNA sequence comparison, the rabbit FcRn α-chain amino acid sequence shows 99% similarity to the rabbit FcRn deposited earlier in GenBank (NM_001122937.1) with two substitutes. The first one is in the α3-domain; Ser189 in our rabbit FcRn sequence (similarly to its human and bovine orthologous) while NM_001122937.1 sequence contains a Pro189 in this position. The other one is in the transmembrane domain; Pro294 in our rabbit FcRn sequence while NM_001122937.1 sequence contains an Ala294 in this position (similarly to its human and bovine orthologous). No functional features have been allocated to these regions thus far. The rabbit FcRn α-chain amino acid sequence also shows high similarity (70–75%) to the coding region of the human, swine, canine, dromedary, bovine, ovine; a moderate homology (63%) to rat and mouse; and a low homology (46%) to possum FcRn sequences querying the Reference protein database with Blastp (NCBI).

**Figure 2:**
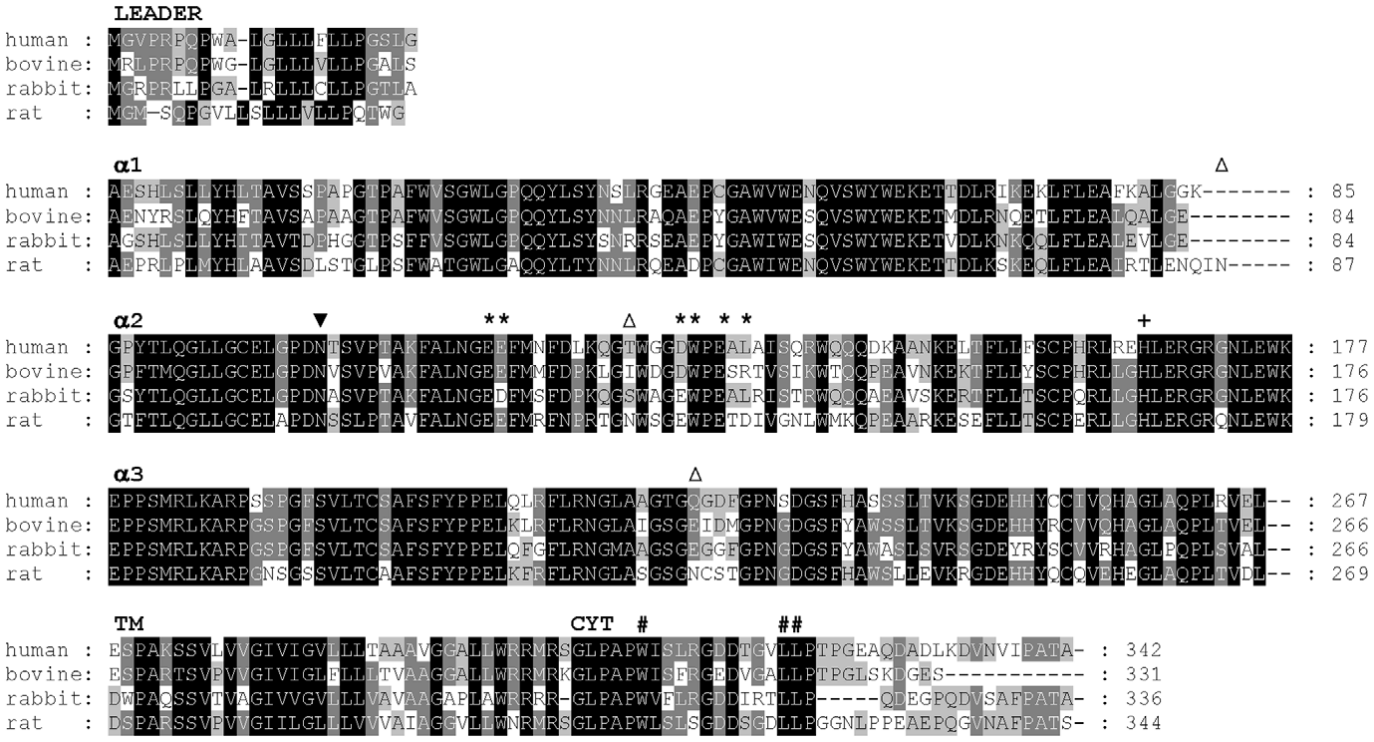
Domain-by-domain alignment of the predicted amino acid sequences for rabbit, human, bovine, rat FcRn α-chain sequences. Structural and functional features are highlighted, potential N-linked glycosylation sites (N-X-S or N-X-T, where X is any amino acid except proline) at positions 87, 128, 225 (present in rat FcRn [9]) and 104 (present in all FcRn species) are indicated by empty and filled triangles to denote non-conserved and conserved sites, respectively. Numbering is based on the rat FcRn sequence. Residues at the interface between rat FcRn and Fc based on a crystallography analysis of a rat FcRn-heterodimeric Fc complex [33] are labelled with asterisks. Conserved His at position 166 is considered to bind to albumin [40] and is indicated by a plus sign. FcRn has been shown to have two endocytosis signals, a tryptophan based motif (W311) and a dileucine motif (L322 and L323) indicated by **#** characters) [41]. Consensus residues are assigned based on the number of occurrences of the character in the column, emphasizing the degree of conservation. The higher the conservation in a column the darker the background of the character [68].

A comparison between rat, mouse, human and bovine FcRn α-chain and β2m residues which are supposed to be involved in binding to IgG molecules have been extensively analyzed based on a crystallography analysis of a rat FcRn-heterodimeric Fc complex [33]. This study compared important residues in the interaction and found that the rat Glu117, Glu118, Glu132, Trp133, Glu135 and Asp137 (aa numbering follows the rat sequence) are highly conserved in mouse, human and bovine and their mutations result in significant loss of binding affinity. By comparing the rabbit FcRn α-chain sequences to its rat [9], human [34], bovine [35] orthologous in **Fig. 2**, we found that among the indicated ones, Glu118 is replaced by Asp118 (conservative substitution) in rabbit while Glu132 is replaced by Asp132 (conservative substitution) in human and bovine. Although, Asp137 (acidic) was proven as important in rat FcRn/IgG interaction, it is not conserved, as the human, orangutan, macaque, rabbit and possum FcRn sequences have Leu (neutral) and the bovine, ovine, swine and dromedary FcRn sequences have Arg (basic) at this position [36]. The rat FcRn Asp137 interacts with His436 of IgG, however, His436 is not conserved in all Fcγ chains and it is possible that human FcRn Leu137 weakly interacts with Tyr436 of human IgG [37]. As rabbit FcRn has Leu137 and rabbit IgG contains Tyr436, these residues are also potentially involved in the interaction of rabbit FcRn – IgG.

The other key residues in IgG that are in contact with FcRn, the Gly191, Ile253, His310 and His435 [33], [38] are highly conserved across species including the rabbit.

FcRn is composed of the FcRn α-chain and the β2m, and the first amino acid of the β2m is also in contact with IgG [33]. In most of the species analyzed so far this is Ile1, and rabbit has a conservative substitution Val1 in this position [39]. The conserved amino acid residues that are involved in FcRn – IgG interaction in rabbit indicate that the two pH-dependent salt bridges involving amino acid residues His310 and His435 of the IgG/Fc would still form at pH 6.0.

FcRn binds not only IgG, but also albumin and the contact residue for this binding in the FcRn is His166 [40] which is conserved and present in all FcRn α-chain sequences thus far characterized, including the rabbit, suggesting that the rabbit FcRn also binds albumin.

The cytosolic tail motifs of rat FcRn that regulate endocytosis and basolateral targeting have been identified by analyses of mutated FcRn variants in transfected IMCD cells [41], [42], [43]. Both tryptophan (W311; with tryptophan replacing the more common tyrosine in the YXXtheta motif) and dileucine (Leu322Leu323) motifs have been shown to play partially redundant roles in endocytosis [41]. The tryptophan and dileucine motifs are conserved across species, including rabbit, suggesting that additional differences such as variations in glycosylation patterns account for cross-species variability in trafficking [44]. There are potential N-linked glycosylation sites (N-X-S or N-X-T; where X is any amino acid except proline) at positions 87 (present in mouse and rat sequences), 104 (present in all FcRn species, including rabbit), 128 (present in mouse and rat sequences), and 225 (present in rat and mouse FcRn sequences).

It is worth to note that some species (e.g., possum, cows, sheep, dromedaries, pigs, and dogs) have cytosolic tails that are 10 residues shorter than those of other species (e.g., humans, macaques, orangutans, rats, and mice). Although, the C-terminal of the cytoplasmic domain of the rabbit FcRn is similar to its human or rat orthologous, there is a five amino acid deletion in this segment (**Fig. 2**). No functional features have been allocated to this deleted region.

**Figure 3:**
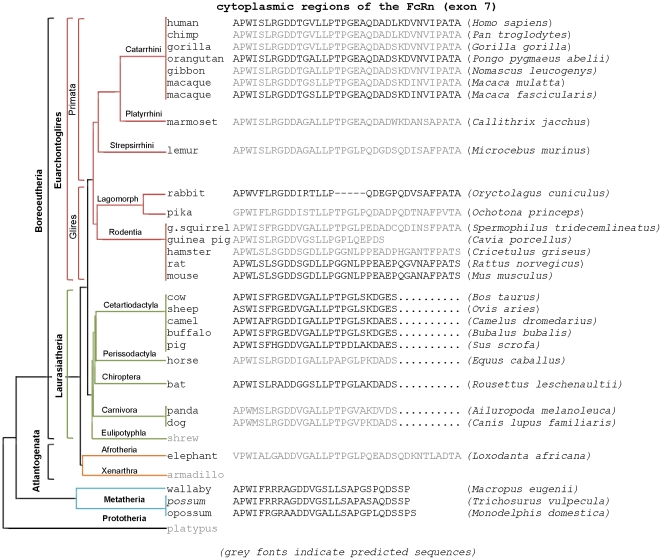
Cytoplasmic domains of the FcRn sequences most likely reflect their phylogenetic position. Marsupials (possum, opossum, and wallaby) have relatively short cytoplasmic domains composed of 27–28 amino acid residues. The next phylogenetic step resulted in clades Atlantogenata and Boreoeutheria. The only (predicted) sequence we found belonging to Atlantogenata (elephant) shows a 7–8 amino acid longer cytoplasmic domain as compared to Marsupials. Boreoeutheria is composed of the sister taxa Laurasiatheria and Euarchontoglires. Species belong to Euarchontoglires analyzed so far (human, chimp, gorilla, orangutan, gibbon, rhesus, marmoset, lemur, rabbit, pika, squirrel, hamster, rat and mouse) preserved these extra amino acids of the FcRn C-terminal with the exception of the guinea pig (based on its predicted amino acid sequence). Rabbit lost five amino acids in a more N-terminal (or middle) part of the cytoplasmic domain. As pika (Ochotona), another Lagomorphs, possesses these residues, the five amino acid deletion is thus specific of either rabbit or Leporidae family. Animals belong to the Laurasiatheria clade (bovine, sheep, pig, horse, bat, dog and panda) lost 10 amino acids of their FcRn C-terminals. Phylogenetic tree was created based on Prasad et al. [45] where some branch lengths were optimized for clarity and space.

Searches of the NCBI and Ensembl databases resulted in finding several FcRn orthologs (beyond the well characterized FcRn sequences) which suggested that the sequences of their cytoplasmic domains most likely reflect the phylogenetic position of these species [45] (**Fig. 3**). Marsupials (possum, opossum, and wallaby) have relatively short cytoplasmic domains that composed of 27–28 amino acid residues. Early mammalian phylogeny resulted in clades Atlantogenata and Boreoeutheria. The only (predicted) sequence we found belonging to Atlantogenata (elephant) shows a 7–8 amino acid longer cytoplasmic domain as compared to Marsupials. Boreoeutheria is composed of the sister taxa Laurasiatheria and Euarchontoglires. Species belong to Euarchontoglires analyzed so far (human, chimp, orangutan, gibbon, macaques, marmoset, lemur, rabbit, pika, squirrel, hamster, rat and mouse) preserved the extra amino acids of the FcRn C-terminal with the exception of the guinea pig (based on its predicted amino acid sequence). Rabbit lost five amino acids in a more N-terminal (or middle) part of the cytoplasmic domain. As pika (Ochotona), another Lagomorphs, possesses these residues, the five amino acid deletion is thus specific of either rabbit or Leporidae family.

Animals belong to the Laurasiatheria clade (bovine, sheep, pig, horse, bat, dog and panda) lost 10 amino acids of their FcRn C-terminals (**Fig. 3**). We have previously analyzed the bovine FcRn sequence and found that the reason of this shorter cytoplasmic domain is a single mutation in the reading frame of the bovine FcRn that resulted in a stop codon. Nucleotides which follow this stop signal represent codons for similar amino acid residues which are found at the 3′-end of the human, rat and mouse molecules [35]. Analyzing the coding sequence of other species in this group shows similar pattern (data not shown).

### 4. Chicken polyclonal antibody raised against the bovine FcRn α-chain cross-reacts with rabbit FcRn α-chain

**Figure 4:**
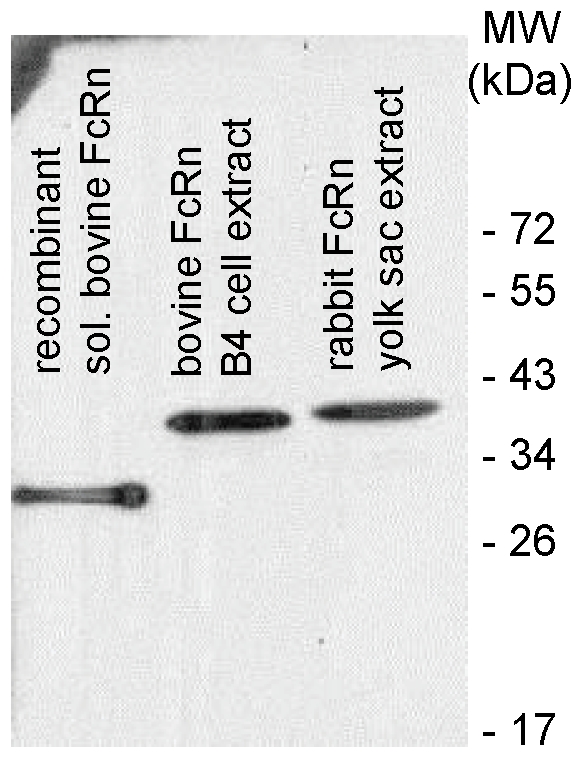
Detection of the rabbit FcRn in Western blot. This Western blot shows that the chicken antibody strongly and specifically reacted with the soluble recombinant bovine FcRn which was used for immunization (its estimated molecular weight is 30 kDa), a ∼40-kDa band that is consistent with the known molecular weight of the bovine FcRn α-chain in the protein extract from a bovine FcRn stably transfected cell line (B4) [46] which strongly expresses the functional form of the bovine FcRn and a ∼40-kDa band that is consistent with the calculated molecular weight of the rabbit FcRn α-chain in the protein extract from the rabbit yolk sac from 24 dpc fetuses.

In order to analyze the rabbit FcRn localization in different tissues and characterize its pH dependent IgG binding, we first tested a polyclonal chicken antibody that was raised against a recombinant, soluble bovine FcRn, if it cross-reacts with the rabbit FcRn in Western blot. **Fig. 4** shows that the chicken antibody strongly and specifically reacted with the soluble recombinant bovine FcRn which was used for immunization (its estimated molecular weight is 30 kDa), a ∼40-kDa band that is consistent with the known molecular weight of the bovine FcRn α-chain in the protein extract from a bovine FcRn stably transfected cell line (B4) [46] which strongly expresses the functional form of the bovine FcRn and a ∼40-kDa band that is consistent with the calculated molecular weight of the rabbit FcRn α-chain in the protein extract from the rabbit yolk sac. Thus, we concluded that the chicken polyclonal antibody that was raised against the bovine FcRn cross-reacts with the rabbit FcRn α-chain.

### 5. Immunohistochemical detection of the rabbit FcRn α-chain in rabbit yolk sac, amnion and placenta

**Figure 5:**
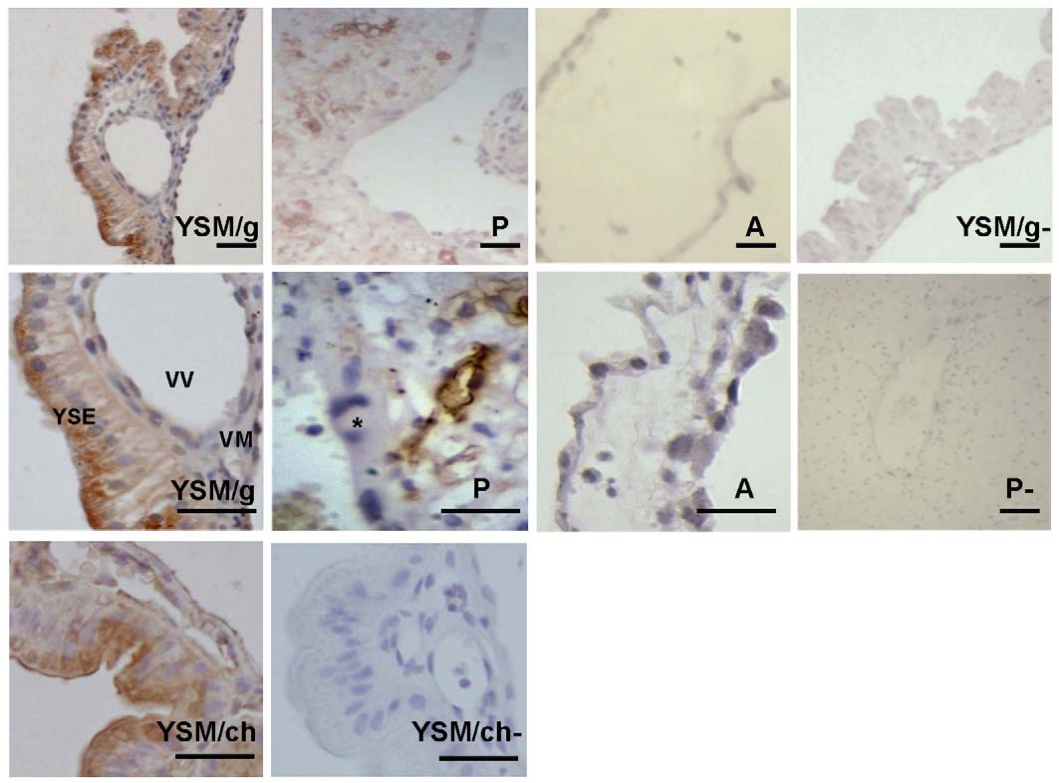
FcRn expression was evaluated in tissue sections from rabbit yolk sac, amnion and placenta from 23 dpc fetuses. For immunohistochemical staining we used a commercially available goat polyclonal mouse FcRn α-chain specific antibody (/g) which cross-reacts with rat, human and bovine FcRn. In the yolk sac membrane (YSM), we also used the chicken FcRn α-chain specific antibody (/ch) which we generated and validated to be rabbit FcRn α-chain specific. We detected strong FcRn specific staining in the apical plasma membrane of the brush border, in the apical region and in vesicles that transverse the endoderm cells (YSE), in the endothelial cells of the vitelline vessels (VV), but not in the vascular mesenchyme (VM) of the rabbit YSM, with the goat and chicken FcRn specific antibodies (**YSM/g** and **YSM/ch**, respectively). In the placenta (**P**), capillary endothelial cells were FcRn α-chain positive, while the trophoblast cells (asterisk) showed no FcRn reactivity. We could not detect the FcRn in the amnion (**A**). (YSM/g-; YSM/ch- and P- represents controls without FcRn α-chain specific antibody; scale bar = 25 µm.)

Previous investigations in the rabbit demonstrated that the transfer of IgG (and a lower extent albumin) occurs across the fetal yolk sac membrane (YSM) from the maternal uterine lumen to the fetus [47]. Since we have successfully detected rabbit FcRn in the rabbit yolk sac (while studying the pH dependent rabbit FcRn IgG interaction), we decided to study the cellular distribution of this receptor in the rabbit yolk sac membrane. FcRn expression was evaluated in tissue sections from YSM, amnion and placenta from 23 dpc fetuses. We have selected this time point from previous data which indicated that the rate of transmission of rabbit IgG injected into the uterine cavity increases nearly linearly from a low level at 20 dpc to a maximum at 26 dpc and declines thereafter [47]. For immunohistochemical staining we used the chicken FcRn α-chain specific antibody we generated against the bovine FcRn and validated to be bovine and rabbit FcRn α-chain specific in Western blot. In parallel, we also used a commercially available goat polyclonal mouse FcRn α-chain specific antibody that cross-reacts with rat, human and bovine FcRn (based on product information). We detected strong FcRn staining in the apical plasma membrane of the brush border's endodermal cells, in the apical region and in vesicles that transverse the endoderm cells of the rabbit YSM with both FcRn specific antibodies (**Fig. 5** **YSM/g and YSM/ch**). The vascular mesenchyme (VM) was not FcRn positive; although the chicken antibody reacted with the endothelial cells of the vitelline vessels (VV) the goat antibody only weakly stained in these cells (**Fig. 5** **YSM/g**). These staining show very similar pattern to those previous studies when absorbed IgG was detected in this tissue [21], [48], [49]. In the placenta, only capillary endothelial cells were FcRn α-chain positive; the trophoblast cells showed no FcRn staining (**Fig. 5** **P**). We could not detect FcRn expression in the amnion (**Fig. 5** **A**). These findings correlate with previous studies and thus we could confirm that the antibodies we used stained the rabbit FcRn in the YSM and also in the placental capillary endothelial cells.

### 6. pH dependent binding of the rabbit FcRn to IgG

**Figure 6:**
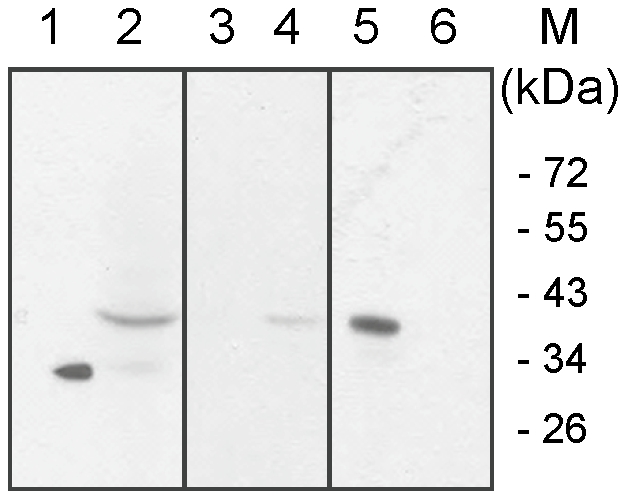
Detection of pH-dependent FcRn binding of IgG in rabbit yolk sac samples. In this assay we analyzed the pH dependent binding of the lysate of rabbit yolk sac of 24 days post coitum (dpc) embryos at pH 6.0 and pH 7.4 to rabbit IgG that was coupled to a Sepharose matrix. Absorbed proteins were then eluated from the matrix and the presence of the FcRn was Western blot tested in these samples as well as in the unbound fractions. The eluted samples contain FcRn only if binding occurred at pH 6 but not at pH 7.4 (bands 5 and 6, respectively). Confirming this result, we could not detect FcRn in the unbound fraction when binding occurred at pH 6.0 (i.e. all FcRn molecule bound to matrix) but FcRn remained in the unbound fraction when the pH of reaction media was neutral (i.e. at this pH no FcRn bound to the IgG-matrix) (bands 3 and 4, respectively). Recombinant bovine FcRn [46] and rabbit yolk sac protein extract were used as positive controls (bands 1 and 2, respectively).

The sequence data of the rabbit FcRn and IgG suggests that their interaction is potentially pH dependent as is the case in all species thus far analyzed [10]. To confirm this hypothesis we analyzed this interaction in a pH dependent IgG-binding assay that was described earlier [50]. In this assay we extracted protein from yolk sac of 24 dpc rabbit fetuses and analyzed its pH dependent binding at pH 6.0 and pH 7.4 to rabbit IgG that was coupled to a Sepharose matrix. Absorbed proteins were then eluted from the matrix and the presence of the FcRn was Western blot tested in these samples as well as in the unbound fractions. **Fig. 6** shows that the eluted samples contain FcRn only if binding occurred at pH 6.0 but not at pH 7.4 (bands 5 and 6, respectively). Confirming this result, we could not detect FcRn in the unbound fraction when binding occurred at pH 6.0 (i.e. all FcRn molecule bound to matrix) but there was detectable FcRn when pH 7.4 buffer was used (i.e. at this pH no FcRn bound to the IgG-matrix) (bands 3 and 4, respectively). Recombinant bovine FcRn [46] and rabbit yolk sac protein extract were used as positive controls (bands 1 and 2, respectively). This study showed that similar to all of its orthologous analyzed so far, the rabbit FcRn interacted with rabbit IgG at pH 6.0, but not at pH 7.4.

### 7. BAC transgenic rabbits carrying and overexpressing extra copies of rabbit FcRn

262E02, a rabbit BAC clone containing the FcRn α-chain coding sequence (FCGRT) located on a 110 kb genomic insert was identified using PCR screening method. Both in human and mouse, the genes are in the same order on the chromosome (synteny): RPL13A, FCGRT, reticulocalbin 3, NOSIP and prolin rich Gla2-like (PRRG2). In OryCun2.0 assembly (Ensembl), RPL13A, FCGRT, reticulocalbin 3 and NOSIP are on the same contig in this order, PRRG2 is also on this contig outside NOSIP but non-annotated. The presence of the ribosomal protein L13a-like, reticulocalbin 3, prolin rich Gla 2-like genes were also identified by PCR on this BAC clone (data not shown). These PCR results and the data we generated with the transgenic rabbits carrying this BAC clone, including extra copies of the integrated rabbit FcRn gene, the higher level of the rabbit FcRn expression and different immunophenotype suggest that the FCGRT gene is fully intact and localized in the internal part of this BAC clone.

To investigate the potential of the FcRn overexpression in transgenic rabbits, the linearized BAC clone harboring the rabbit FCGRT gene and its regulatory region was microinjected into fertilized rabbit zygotes. 1724 injected embryos were transferred into 95 pseudopregnant females. Altogether 125 rabbits were born from 29 does. Transfer and transgenic efficiencies were lower in comparison to our earlier transgenic rabbit experiments [51], [52], which we hypothesize to be caused by the relatively large size of the transgene. Five transgenic founders were identified by BAC backbone specific PCR primers, one of which was stillborn. Three transgenic lines were originally established carrying the transgene. We selected one transgenic line - #78 - to further characterize its immunophenotype.

**Figure 7:**
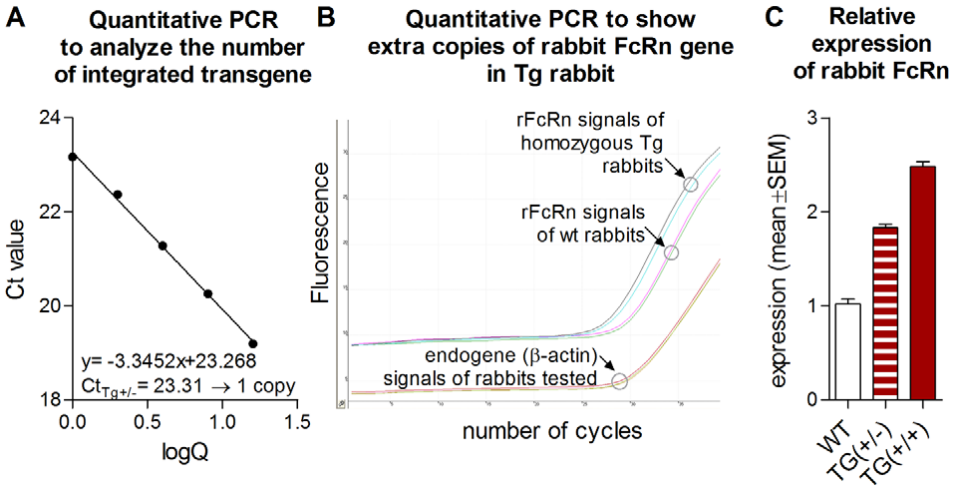
Quantitative real time PCR analysis of transgenic rabbit line #78. We determined the copy number of the transgene integrated into the genome and its expression levels by real time PCR. **A.** Quantitative PCR standard curve and equation to determine the exact copy number of the transgene. According to the linear regression calculation, the 23.31 Ct value that represents the hemizygous line #78 (TG +/−) results in 0.97 which corresponds a single copy integration event (Q represents copy number). **B.** We also detected the integrated extra copies of the rabbit FcRn gene in homozygous Tg rabbits by quantitative genomic PCR. **C.** Relative rabbit FcRn expression levels in hemizygous and homozygous transgenic line #78 shows expression of the transgene in leukocytes of the animals tested. Values shown are the mean ± SEM. TG (+/−)- transgenic hemizygous, TG (+/+)- transgenic homozygous, WT- control animals.

BAC copy number was determined by absolute quantification of the transgene by real time PCR and found that one and two extra copies of the transgene had been integrated in hemizygous and homozygous Tg rabbits, respectively (**Fig. 7A**). We also detected the integrated extra copies of the rabbit FcRn gene in homozygous Tg rabbits by quantitative genomic PCR (**Fig. 7B**).

To distinguish between the rabbit FcRn endogene and transgene expression, a quantitative real time RT-PCR assay was established. Rabbit FcRn levels were normalized to rabbit beta-actin. RNA was isolated and pooled from the leukocytes of 5-5-4 homozygous, hemizygous and control animals, respectively. We detected elevated levels in the leucocytes of the transgenic animals, as it showed 1.83-fold and 2.49-fold higher expression of the rabbit FcRn mRNA in hemizygous and homozygous animals, respectively, than those of the wild-type rabbits (**Fig. 7C**).

In conclusion, our data show that the integrated transgene is expressed as the rabbit FcRn α-chain mRNA level is higher in both the hemizygous and homozygous rabbits as compared to their wt controls. In addition there is a difference between the hemizygous and heterozygous animals.

### 8. Increased serum persistence of the rabbit IgG in transgenic rabbits

**Figure 8:**
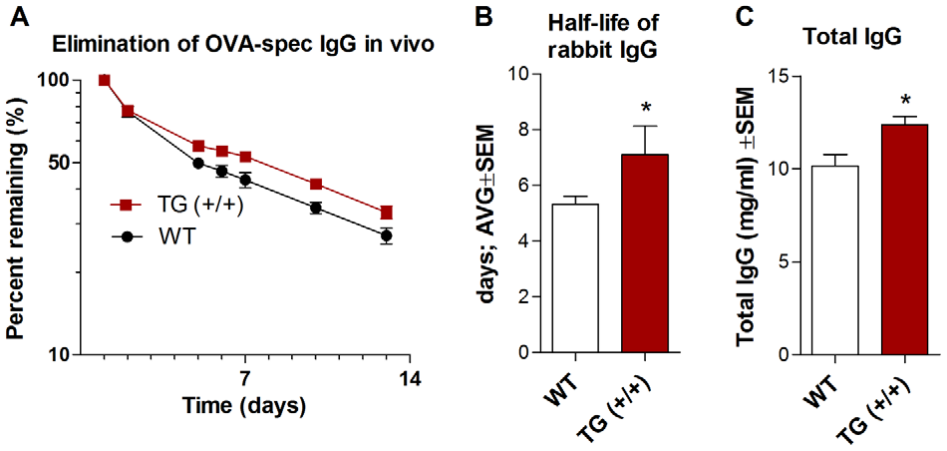
Reduced IgG catabolism in Tg rabbits that carry two extra copies of the rabbit FcRn. We have analyzed the half-life of rabbit IgG in Tg (homozygous; +/+) and wt rabbits from days 2–13 after injecting OVA-specific rabbit IgG i.v. into these animals. **A–B.** Our analysis showed that the Tg rabbits have increased serum persistence of rabbit IgG as the beta phase half-lives of the IgG were 7.1±0.5 days (mean ± SEM) as compared to their controls which showed 5.3±0.3 days. **C.** This difference may be even greater as non-immunized Tg rabbits have higher total IgG levels as compared to their controls. Values shown are the mean ± SEM. (*, *P*<0.05). The experiment was repeated two times with similar results.

**Figure 9:**
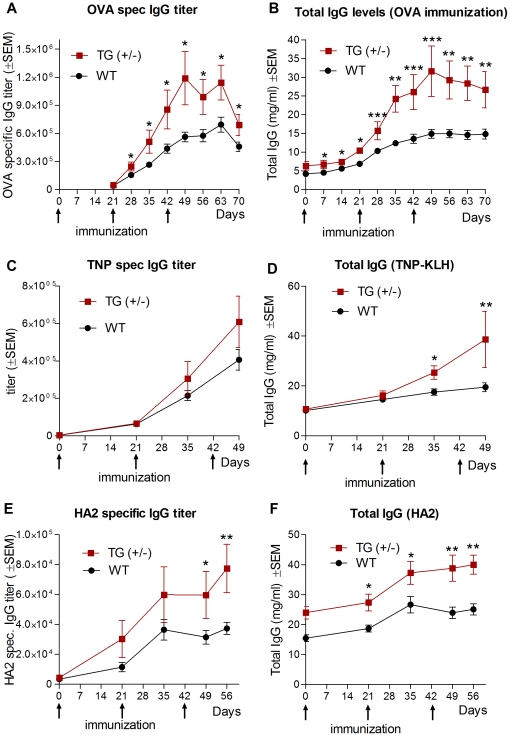
Augmented humoral immune response in transgenic rabbits. **A–B**. Tg+/− and wt rabbits (4 and 8 animals, respectively) were immunized with OVA. After the booster immunization the OVA-specific IgG titers were nearly double in Tg rabbits as compared with the wt animals. We found that the total IgG levels rose steadily after immunization and reached peak levels on day 49 in Tg and wt animals. **C–D**. Tg+/− and wt rabbits (5 and 5, respectively) were immunized with TNP-BSA. The mean TNP-specific IgG level was higher in Tg rabbits as compared to their wt controls at the peak of the immune response, on day 49, one week after the second booster immunization with an almost doubled level of total IgG as compared to their wt controls. **E–F**. The same rabbits which had been immunized with TNP-BSA, were immunized with a conserved influenza hemagglutinin epitope (HA2) conjugated to KLH. The mean of the HA2-specific IgG levels was double at the peak of the immune response, on day 56, two weeks after the second booster immunization, with an almost doubled level of total IgG as compared to their wt controls. Values shown are the means ± SEM. (*, *P*<0.05; **, *P*<0.01; ***, *P*<0.001.).

Pharmacokinetic studies in mice overexpressing FcRn showed that the efficiency of IgG protection was higher [14], [27], [53]. In order to analyze if FcRn overexpression results in a similar reduction of IgG catabolism in rabbits, we analyzed the pharmacokinetic behavior of rabbit IgG in Tg (+/+) animals that carry two extra copies of the rabbit FcRn and compared these results with wt rabbits. The clearance curves of the measured OVA-specific IgG were biphasic, with phase 1 (alpha phase) representing equilibration between the intravascular and extravascular compartments, and phase 2 (beta-phase) representing a slow elimination. We analyzed the beta phase half-lives of rabbit IgG from day 2 to 13, and found that the Tg rabbits demonstrated increased serum persistence of rabbit IgG because the beta phase half-lives were 7.1±0.46 days (mean ± SEM) as compared to their controls which showed 5.3±0.3 days (**Fig. 8A, 8B**). The difference of the beta-phase half-life may be even greater as Tg animals have higher total IgG level (**Fig. 8C** **,** **Fig. 9B**) as compared to the controls, which increases the fractional catabolic rate. As a result, we concluded that the transgenic FcRn was functionally expressed and elongated the half-life of rabbit IgG in these animals.

### 9. Augmented humoral immune response in transgenic rabbits

To investigate the consequences on the humoral immune response in transgenic rabbits that overexpress FcRn, we first immunized these animals (Tg +/− carrying one extra copy) with OVA. No difference between Tg and wt animals was observed during the primary immune response; however, after the booster immunization, the OVA-specific IgG titers were nearly doubled in Tg rabbits as compared with the wt animals and this difference was significant (*P*<0.05) (**Fig. 9A**). We found that the total IgG level rose steadily after immunization and reached peak levels on day 49 in both Tg and wt animals. Notably, we found a remarkable and significant difference at the highest IgG levels, which were 31.61±2.7 mg/ml in Tg rabbits versus 14.8±2.6 mg/ml (mean ± SEM) in wt (*P*<0.01) (**Fig. 9B**).

Following this experiment, we analyzed a hapten-specific immune response by immunizing Tg (+/−) and wt rabbits with TNP-BSA. The TNP-specific IgG levels were modestly higher in Tg rabbits (with the exception of one Tg animal that showed double TNP-specific IgG) as compared to their wt controls at the peak of the immune response, on day 49, which was one week after the second booster immunization (**Fig. 9C**). The total IgG level was also significantly (*P*<0.01) elevated in the Tg animals compared to their controls (**Fig. 9D**).

Finally, the same rabbits that had been immunized by TNP-BSA were immunized with a conserved influenza hemagglutinin epitope (HA2; which is considered as a weakly immunogenic antigen) conjugated to KLH. (We used these rabbits due to the limitation in the number of Tg animals.) The mean of the HA2-specific IgG levels was double (*P*<0.01) at the peak of the immune response, on day 56, which was two weeks after the second booster immunization, with an almost doubled level of total IgG as compared to their wt controls (**Fig. 9E, 9F**).

In conclusion, Tg rabbits that overexpress rabbit FcRn show augmented humoral immune response, similarly to the result shown by those Tg mice that overexpress bovine FcRn [28], [29], [52].

## Discussion

Immunization protocols for the production and subsequent maintenance of high levels of antigen-specific polyclonal antibody require hyperimmunization. Although serum IgG levels may exceed normal levels following immunization, the rate of IgG breakdown is also exponentially increased [2], [22]. Therefore, frequent immunizations are required to maintain high levels of Ag-specific IgG. FcRn is known to be involved in transporting IgGs within and across the cells of diverse origin, and in doing so, they regulate IgG and albumin concentrations and transport throughout the body [54]. More recently, several publications have shown that FcRn plays major roles in Ag-IgG immune-complex phagocytosis by neutrophils [15], in antigen presentation of IgG immune complexes by professional antigen presenting cells [16], [17], [18], [19] and in generating antigen specific antibodies [18]. We, and others, have shown that higher than normal expression levels of FcRn reduced exogenous IgG catabolism in Tg mice, resulting in higher circulating levels of IgG [14], [26], [27]. Our more recent studies have demonstrated that FcRn overexpression in Tg mice greatly augment humoral immune response [28], [29], [55].

The first step of our study was to clone the full-length cDNA of the rabbit FcRn α-chain by using 5′- and 3′-RACE PCR technology, based on available rabbit FcRn fragments. Our data (together with another rabbit FcRn α-chain sequence in the meanwhile deposited into GenBank, without further characterization) shows that the rabbit FcRn α-chain amino acid sequence shows high similarity to its orthologous (**Fig. 2**). One of the hallmarks of the Fc - FcRn binding interaction is its pH dependence; Fc has full binding activity towards FcRn at pH 6, but minimal binding occurs at pH 7.4. Previous experimental data showed, that gain of significant binding activity at near neutral pH results in reduced release during exocytosis at the plasma membrane and enhanced trafficking of the IgG into lysosomes [56]. Structural data [33], [57] and other site-directed mutational studies [58] implicated Glu115 and Asp130 on human FcRn (Glu117 and Glu132 in rat FcRn) as being responsible for the pH dependent binding to residues His310 and His435 on Fc. A proton on histidine's imidazole side-chain can be titrated between pH 6 and 7.4 and results in an ionic molecular switch for binding to FcRn. We found that the key residues that are involved in the FcRn - IgG interaction are highly conserved in rabbit, suggesting that the binding occurs at pH 6.0, like in other species. To confirm this hypothesis, we analyzed this interaction in a pH dependent IgG-binding assay that was described earlier [50]. We have used yolk sac lysates of 24 dpc fetuses and analyzed its pH dependent binding at pH 6.0 and pH 7.4 to rabbit IgG that was coupled to a Sepharose matrix. Absorbed proteins were then eluated from the matrix and the presence of the FcRn was Western blot tested in these samples as well as in the unbound fractions. **Fig. 6** shows that the eluted samples contain FcRn only if binding occurred at pH 6.0, but not at pH 7.4, indicating that, similar to all of its orthologous analyzed so far, the rabbit FcRn interacts with rabbit IgG at pH 6.0, but not at pH 7.4. The strict pH dependent interaction of IgG and the rabbit Fc receptor expressed in the rabbit yolk sac was questioned by Meads and Wild [49]. In their study, they used a similar pH dependent binding assay in which pH dependent interaction of matrix linked rabbit IgG and soluble protein extract from yolk sac endoderm cells of 26 dpc fetuses was tested. After binding, they fractionated the eluted samples by SDS-PAGE; however, they could not detect specific band when binding occurred at pH 6.0 followed by elution at pH 8.0, using silver-staining. We believe that the FcRn specific antibody we used (in a more specific Western blot) made the FcRn specific detection possible in our system.

It has been well established that rabbit placenta is not permeable to maternal IgG and that the transfer of this antibody (and a lower extent albumin) occurs across the rabbit fetal yolk sac membrane (YSM) from the maternal uterine lumen to the fetus [47]. Because we have successfully detected rabbit FcRn expression in rabbit fetuses (**Fig. 1** and **Fig. 4**), and also in yolk sac lysates (while studying the pH dependent rabbit FcRn IgG interaction; **Fig. 6**), we then analyzed the cellular distribution of this receptor in the rabbit yolk sac membrane, amnion and placenta from 23 dpc fetuses. We have selected this time point from previous data which indicated that the rate of transmission of rabbit IgG injected into the uterine cavity increases nearly linearly from a low level at 20 dpc to a maximum at 26 dpc and declines thereafter [47]. We detected strong FcRn staining in the apical plasma membrane of the brush border's endodermal cells, in the apical region and in vesicles that transverse the endoderm cells of the rabbit YSM with both FcRn specific antibodies, but there was no staining in the placental trophoblast cells or in the amnion (**Fig. 5**). Our immunohistochemistry data show a very similar pattern to those previous studies (when absorbed IgG was detected in these tissues [21], [48], [49]), suggesting that we detected rabbit FcRn in the YSM, which is responsible for the maternal IgG transport.

Beyond the pH dependent binding assay, Meads and Wild analyzed the IgG transport in an in vitro culture system using rabbit visceral yolk sac and found that monensin did not prevent selective transcytosis of human IgG, suggesting that an acidic compartment may not be needed for transcytosis [49]. To further clarify the role of FcRn in the maternal IgG transport, we are currently analyzing if there is a more efficient maternal IgG transport in our Tg rabbits that overexpress FcRn. It is worth to mention, that maternal IgG transport in rabbits has recently gained a new wave of interest for its usefulness for analyzing the effect of therapeutic monoclonal antibodies or Fc-fusion proteins on embryofetal development [59], [60].

Having characterized the rabbit FcRn, we were particularly interested in generating Tg rabbits that overexpress FcRn and study their IgG protection as well as humoral immune response. We have used BAC transgenesis and created several Tg rabbit lines that carried the integrated BAC clone and the rabbit FCGRT gene that encodes the rabbit FcRn. After initial characterization, line #78, which carries one extra copy when hemizygous or two extra copies when homozygous (**Fig. 7A**), was selected for further analysis. FcRn is known to be expressed in bone marrow derived cells [10], and thus we used peripheral blood leucocytes to analyze the expressional level of the rabbit FcRn in these animals. Our quantitative PCR analysis showed overexpression of this receptor in hemizygous and homozygous Tg animals compared to wt controls (**Fig. 7B**). This result was similar to our Tg mice that overexpress bovine FcRn and were also created by BAC transgenesis [27].

We then analyzed the IgG clearance in these Tg rabbits and found that transgenic animals that overexpress rabbit FcRn protected more efficiently the exogenous IgG as compared to their controls (**Fig. 8**). This data confirmed that the transgenic rabbit FcRn is functional and that FcRn overexpression results in longer IgG half-life, as we, and others, have previously shown in mice [14], [26], [27]. Of greatest interest was whether better protection of IgG in FcRn Tg rabbits results in increased levels of Ag-specific antibodies following immunization. Using these Tg rabbits we demonstrated that immunization with ovalbumin (OVA), TNP haptenated protein and an influenza hemagglutinin peptide generated higher levels of Ag-specific IgG titers and also higher total IgG levels as compared to their controls (**Fig. 9**). Again, these data showed that FcRn overexpression has similarly enhancing effect on the humoral immune response in rabbits as we observed in mice [29], [55], [61]. We are currently analyzing the number of antigen specific B-cells, the cellular composition of the spleen and other secondary lymphoid organs during immunization that we demonstrated contribute in this positive effect in Tg mice that overexpress FcRn [31].

Like mice, rabbits have a short generation time, produce large numbers of offspring and can be raised under specific pathogen free conditions. They have long been an important source of polyclonal and more recently also monoclonal antibodies [62], [63]. As a result, there is an increasing interest in raising human antibodies in these animals. The inactivation of immunoglobulin genes in pigs was reported [64], [65]; however, the inactivation of endogenous immunoglobulin genes in combination with the addition of human immunoglobulin genes has so far only been achieved in mice and cattle [66]. Experiments are now underway to achieve this in rabbits [67]. The responsiveness of these animals to any given antigen, and subsequent production of sufficient quantities of pAbs, is a key prerequisite for the viability of these systems for commercial use. Human IgG injected into the maternal circulation is transported well to the rabbit fetus [47] and its clearance is similar to rabbit IgG [3] suggesting that rabbit FcRn binds efficiently human IgG. Thus, the FcRn overexpression technology could make qualitative and quantitative contributions to the efficiency of these engineered animals, too.

## Materials and Methods

### Ethics statement

All the treatments of animals (rabbits) in this research followed by the guideline of the Institutional Animal Care and Ethics Committee at Agricultural Biotechnology Center that operated in accordance with permissions 22.1/3507/000/2008 and 22.1/1127/003/2209 issued by the Central Agricultural Office, Hungary or the Institutional Animal Care and Ethics Committee at ImmunoGenes Ltd. that operated in accordance with permission 22.1/601/000/2009 issued by the Central Agricultural Office, Hungary.

### Cloning and sequencing the full length of the rabbit FcRn cDNA

To obtain the complete rabbit FcRn cDNA we used rapid amplification of the cDNA ends (RACE) technique based on rabbit specific FcRn fragments that were identified in the BLAST EST database (EB377775.1; DN888548.1. and EB37774.1). Total RNA was extracted by using RNAzol™ B (Tel-Test Inc, Friendswood, TX) from rabbit liver and spleen and then reverse transcribed with SuperScript TM III Reverse Transcriptase (Invitrogen, UK).

**5′-RACE** – The 5′-end of the rabbit FcRn was isolated using the 5′ RACE System for Rapid Amplification of cDNA Ends Version 2.0 Kit (Invitrogen, UK) according to the manufacturer's instruction. Briefly, total RNA was reverse transcribed using a rabbit FcRn-specific oligonucleotide (GSP1: 5′-AAG CCC AGG CGT AGA AGG-3′). After cDNA synthesis, unincorporated dNTPs, primer and proteins were separated from cDNA with gel purification. A homopolymeric tail was then added to the 3′-end of the cDNA using TdT (Terminal deoxynucleotidyl transferase) and dCTP. This product was directly used for a PCR reaction with another rabbit FcRn specific primer (GSP2: 5′- GCT CCT TCC ACT CCA GGT T – 3′) and a deoxyinosine-containing anchor primer (Abridged Anchor primer). This amplicon was then further applied in a nested PCR reaction with another rabbit FcRn specific primer (GSP3: 5′- GCT TGG GGT CGA AAC TCA T-3′) and an adaptor primer to the Abridged Anchor primer (AUAP).

**3′-RACE** - The 3′-end of the rabbit FcRn was isolated using the 3′ RACE protocol. Briefly, total RNA was reverse transcribed using a (dT)_17_-adapter primer (5′- GAC TCG AGT CGA CAT CGA (T)_17_-3′). The resultant cDNA was then subjected to 3′RACE-PCR amplification using the adaptor primer (5′-GAC TCG AGT CGA CAT CG-3′) and a rabbit FcRn specific primer (GSP4: 5′-AAC CTT CCT GCT CAC CTC CT-3′). This amplicon was then further applied in a nested PCR reaction in which the same adaptor primer and another rabbit FcRn specific primer (GSP5: 5′- GCG ACG AGT ACC GCT ACA G-3′) was used.

**Cloning and Sequencing** - Based on the expected size, the proper Taq polymerase generated fragments were cloned into the pGEM-T Easy vector (Promega Corp., Madison, WI) and fully sequenced. FcRn sequences were searched in DDBJ/EMBL/GenBank databases using the BLAST program. Sequence editing, comparisons were accomplished using GeneDoc version 2.7.000 [68].

### Rabbit FcRn α-chain mRNA expression in different tissues and cells

To evaluate tissue specific expression of FcRn, we used rabbit FcRn specific primer pair (R1B1: 5′-CTG AAC GGT GAG GAC TTC AT-3′) and GSP1 primer that resulted in a 380 bp long amplicon. To check the integrity of the synthesized cDNA we also amplified rabbit GAPDH in these samples (RabGAPDHf: 5′- GAG CTG AAC GGG AAA CTC AC-3′ and RabGAPDHfr: 5′-CCC TGT TGC TGT AGC CAA AT-3 primer pair was used that resulted in a 304 bp long amplicon). Rabbit cDNA synthesized from kidney, ovary, uterus, salivary gland, lymph node, lung, brain, liver, spleen, small intestine, placenta, amnion and yolk sac, as well as rabbit blastocyst (3.5 and 4.5 days post coitum; dpc), rabbit embryo (6, 9 and 13 dpc) were utilized in PCR reaction with the primers and RedTaq (Sigma-Aldrich Co., St. Louis, MO, USA). We also analyzed the rabbit FcRn expression in peritoneal macrophages, which were isolated based on a standard protocol [69]. Briefly, 500 ml 3% Brewer thioglycol medium was injected into the peritoneal cavity and the animal was euthanized 4 days later followed by harvesting peritoneal exudate cells (PEC). Purity of the macrophages has been analyzed by FACS, in which the cells were labeled by Alexa647-labeled rat anti-mouse CD11b (eBioscience, San Diego, CA, USA) or Alexa647-labeled rat IgG2b (eBioscience, San Diego, CA, USA), as isotype control, respectively.

### Generation of a chicken polyclonal rabbit FcRn α-chain specific antibody

We used a chicken polyclonal antibody that was generated against a recombinant soluble bovine FcRn protein [46] for Western blots and also in immunohistochemistry. In brief, 200 µg of the recombinant protein (that lacks the transmembrane and cytoplasmic domains of the bovine FcRn α-chain) with complete Freund adjuvant was injected intramuscular (m. pectoralis) and subcutan (neck) in multiple sites in chickens. Three weeks later, the animals were boosted with 100 µg of the recombinant protein with incomplete Freund adjuvant, and this was repeated 4 times until high titer anti-bovine FcRn serum was raised (analyzed by ELISA and Western blot). IgY was then purified from the egg-yolk based on standard protocol [70].

### Immunohistochemical detection of the rabbit FcRn α-chain in rabbit yolk sac, amnion and placenta

For immunohistochemistry, 23 day old fetuses were used. Yolk sac, amnion and placenta were collected, fixed in 4% PFA (Sigma-Aldrich Co., St. Louis, MO, USA) and embedded in paraffin (Sigma-Aldrich Co., St. Louis, MO, USA). Tissue samples were sectioned (4 µm) and placed on silanized slides. After deparaffination, we used 10 mM sodium citrate buffer (pH 6.0), at 95°C for 10 min for heat-induced epitope retrieval, which was followed by endogen peroxidase blocking (1% H_2_O_2_; Fluka Chemie AG, Switzerland). For blocking, 2.5% bovine serum albumin (BSA, Sigma-Aldrich Co., St. Louis, MO, USA) diluted in PBS was used. Sections were incubated with a goat polyclonal mouse FcRn α-chain specific antibody that cross-reacts with rat, human and bovine FcRn (K13; Santa Cruz, USA). We also used the chicken bovine FcRn α-chain specific antibody that we validated to be rabbit FcRn α-chain specific to detect FcRn in rabbit yolk sac membrane. All samples with the primary antibody were incubated at 4°C overnight. After being rinsed with 3× 1% BSA/PBS for 10 min, HRP conjugated donkey anti-goat IgG (Santa Cruz, CA, USA) or HRP conjugated rabbit anti-chicken IgG-Fc (Biodesign International, Saco, ME, USA) as secondary antibody was applied for 1 h at RT. For detection, 0.25 mg/ml DAB (Sigma-Aldrich Co., St. Louis, MO, USA) diluted in PBS at RT was used. Slides were counterstained with Meyer's Hematoxylin (Fluka Chemie AG, Switzerland) and mounted with DPX mounting medium (Fluka Chemie AG, Switzerland). Sections were examined and photographed with an Olympus microscope (Olympus, Hamburg, Germany).

### Analysis of pH dependent binding of the rabbit FcRn to IgG

FcRn-IgG pH dependent binding assay was performed based on a previously described protocol [50] with some modification. As a first step, polyclonal rabbit IgG was coupled to CNBr- activated Sepharose 4B (Pharmacia Fine Chemicals, Uppsala, Sweden), according to the manufacturer's instructions. Groups remaining active were blocked with 1 M ethanolamine, pH 8.0. The matrix was then washed and kept in 0.1 M PBS (pH 6 or 7.4) containing 5 mg/ml CHAPS (Sigma-Aldrich Co., St. Louis, MO, USA).

Yolk sac samples were collected from rabbit fetuses 24 dpc and homogenized for 5000 rpm with a Precellys 24 homogenizer (Bertin Technologies, Montigny-le-Bretonneux, France) with 0.1 M PBS (pH 6.0 or 7.4) and 0.5% CHAPS (Sigma-Aldrich Co., St. Louis, MO, USA). Then, tissue debris was separated by centrifugation for 20 sec, at 13000 rpm, 4°C and the samples were stored frozen (−70°C) until use. Immediately before the IgG binding assay, the samples were thawed and protease inhibitor cocktail (Sigma-Aldrich Co., St. Louis, MO, USA) was added at 20 µl/ml final concentration.

100-100 µl rabbit-IgG-Sepharose 4B matrix and 300-300 µl lysate, in pH 6 or 7.4, respectively, were mixed and incubated overnight at 4°C. As a control, 50 µg recombinant bovine FcRn was also used. Then the matrix was centrifuged (1000 RPM, 5 minutes, 4°C), the supernatant (unbound) was concentrated with 30.000 MW Microcon filter (Millipore, Billerica, MA, USA) and stored frozen in reducing electrophoresis sample buffer. The matrix was then washed three times in 0.1 M PBS (pH 6.0 or 7.4) and 0.5% CHAPS, then the adsorbed proteins were boiled with reducing electrophoresis sample buffer. The eluted proteins were subjected to electrophoresis on 10% SDS-PAGE gel and transferred to a Hybond-P PVDF membrane (Amersham Pharmacia Biotech, Piscataway, NJ, USA). Blots were probed with a 1∶10.000 diluted chicken anti-bovine FcRn antibody according to standard protocol. Bound bovine FcRn α-chain antibody was detected with horseradish peroxidase-conjugated rabbit anti-chicken IgY (Fc) (Biodesign International, Saco, ME, USA) antibody and enhanced chemiluminescence, using ECL-Plus chemiluminescent system (Amersham Pharmacia Biotech, Piscataway, NJ, USA). The recombinant bovine FcRn [46] was used as positive control.

### Selection of the BAC clone that harbors the rabbit FcRn α-chain gene (rabbit FCGRT)

A rabbit BAC library which was constructed from white blood cells of a New Zealand rabbit [71] was used to identify a BAC clone that contains the rabbit FCGRT gene, with rabbit FcRn specific PCR screening method (primers: OCU_FCGRTf: 5′-GGG ACT CCC TCC TTC TTT GT-3′ and OCU_FCGRTr: 5′ AGC ACT TCG AGA GCT TCC AG-3′).

### Generation of the transgenic (Tg) rabbit carrying extra copies of the rabbit FCGRT

BAC clone 262E02 harboring the rabbit FCGRT gene was purified using Qiagen Large-Construct kit (Qiagen GmbH, Germany) and was linearized by PmeI restriction enzyme digestion. Subsequently, the linearized DNA was run on a pulsed-field gel and recovered from the gel by GElase digestion (Epicentre Biotechnologies, Madison, WI, USA) [72]. The linearized BAC DNA was injected into pronuclei of New Zealand White rabbit zygotes. Injected eggs were transferred into pseudopregnant females. Genomic DNA samples of founder rabbits were collected from ear biopsies. The founder rabbits were genotyped by a pair of primers: 5′-CGA AAC AGT CGG GAA AAT CT-3′ and 5′-GGC ATC GTG TGT AAG CAG AA-3′ which are specific for the BAC backbone. Transgenic rabbit lines were maintained by sibling mating.

### Determination of the transgene copy number by real time PCR

To determine the copy number of the BAC construct carrying the rabbit FCGRT transgene integrated into the genome, a quantitative PCR was carried out using primers designed to amplify a 250 bp fragment of the BAC sequence (5′-CGA AAC AGT CGG GAA AAT CT-3′ and 5′-GGC ATC GTG TGT AAG CAG AA-3′). Two-fold dilutions of the purified BAC DNA samples were spiked into 1.5 ng/µl rabbit genomic DNA (final concentration). This created a series of standard samples such that the ratio of BAC molecules ranged from 1 to 16 BAC copies per diploid rabbit genome. We used these samples to generate calibration curve with equation for estimating the copy number from sample rabbit line #78. Amplification was analyzed using the Power SYBR Green PCR Master Mix (Life Technologies, USA) run on Rotorgene RG-3000.

Homozygous transgenic animals were selected using genomic qPCR. qPCR was conducted with 40 ng DNA in a 20 µl reaction mix using the TaqMan PCR Universal Master Mix (Applied Biosystems, USA). Custom TaqMan chemistry assays were used for assaying rabbit β-actin (endogenous control) and rabbit FcRn (Life Technologies, USA and Integrated DNA Technologies, Germany, respectively) run on Rotorgene RG-3000.

### Determination of the rabbit FcRn expression by real time PCR

To test if rabbit FcRn expression increases in Tg rabbits, we setup a real time quantitative RT–PCR, in which RNA was isolated from rabbit leukocytes with the RNeasy Plus Mini kit (that includes a DNase digestion step, Qiagen GmbH, Germany) and first strand of cDNA was synthesized using the High Capacity cDNA Reverse Transcription Kit (Life Technologies, USA). Quantitative PCR was performed with RotorGene RG-3000 and analyzed using ΔΔCt method (RotorGene software, Corbett Research, Sidney, Australia). Power SYBR Green PCR Master Mix (Life Technologies, USA) was applied for quantitative PCR reactions. Rabbit FcRn specific primers were 5′-TTG GAT CTG GGA AAG CCA GGT G-3′ and 5′-TGT TCT TCA GGT CCA CGG TCT C-3′. The expression of FcRn was normalized to rabbit beta-actin amplified by primers 5′-ATC CTG ACG CTC AAG TAC CC-3′ and 5′-AGC TCG TTG TAG AAG GTG TGG T-3′.

### In vivo analysis of the rabbit IgG half-life in transgenic rabbits

Following a pre-bleed, five, age and weight matched Tg (homozygous #78 animals that carry two extra copies of the rabbit FcRn; Tg +/+) and wt siblings as controls, respectively, were injected intravenously (ear vein) with a single injection of 1 mg anti-OVA rabbit IgG in 1 ml of sterile PBS, and blood samples were collected from ear vein during the next 13 days. OVA specific IgG levels in the serum samples were measured by an ELISA assay (as it is described below) and expressed as OD values. The serum concentrations of OVA specific IgGs were presented as percent remaining in the circulation at different time points after injection compared with day 1 values (100%). IgG clearance data was analyzed by fitting the data of days 2–13 to the one-compartmental model using WinNonLin professional, version 5.1 (Pharsight, Mountain View, CA).

### Analyzing the humoral immune response of Tg rabbits overexpressing rabbit FcRn

#### Immunization

In the first experiment, Tg (hemizygous #78 animals that carry one extra copy of the rabbit FcRn; Tg +/−) and wild type (wt) rabbits (3 months old siblings, four and eight Tg and wt, respectively) were intramuscularly immunized with 200 µg OVA in Complete Freund's Adjuvant (CFA) and challenged 21 and 42 days later with 100 µg OVA in Incomplete Freund's Adjuvant (IFA). In the second experiment, Tg +/− and wild type (wt) rabbits (3 months old siblings, five in each group) were intramuscularly immunized with 200 µg TNP-BSA in CFA and challenged 21 and 42 days later with 100 µg TNP-BSA in IFA. Finally, Tg and wt rabbits (7 months old siblings, previously immunized with TNP-BSA) were intramuscularly immunized with a KLH conjugated polypeptide (HA2: N′-TQNAINGITNKVNSVIE-C′) that consists of the highly conserved amino acids 41–57 of the α-helix of the influenza hemagglutinin subunit 2, Influenza A/California/07/09(H1N1) [29]. Animals were immunized with 200 µg HA2 peptide-KLH conjugate (HA2-KLH) in CFA and challenged 21 and 42 days later with 100 µg of the conjugate in IFA.

#### ELISA measurements of antigen-specific and total immunoglobulin levels

High binding ELISA plates (Corning Inc. NY, USA) were coated with 5 µg/ml OVA, 5 µg/ml TNP-IgY, 5 µg/ml HA2-peptide or 1 µg/ml unlabeled goat anti-rabbit polyclonal IgG (H+L) (Southern Biotechnology Associates, Inc., Birmingham, AL, USA), respectively, in 0.1 M sodium carbonate-bicarbonate buffer (pH 9.6) for 2 hours at room temperature and then were washed with 0.1 M phosphate-buffered saline (PBS, pH 7.2) containing 0.05% Tween-20 (PBS-T) and blocked with PBS containing 1% BSA or PBS-T for 1 h at room temperature. Diluted serum samples were added to the wells and incubated for 1 h at room temperature. Each plate included standard controls of pooled antigen-specific immune sera or serially diluted purified rabbit IgG. After washing, bound serum antibody was revealed by horseradish peroxidase (HRP)-labeled goat anti-rabbit IgG (Southern Biotechnology Associates Inc. Birmingham, AL, USA) or by HRP-labeled goat anti-rabbit IgM (Southern Biotechnology Associates Inc. Birmingham, AL, USA). The peroxidase-conjugated antibodies were detected using tetramethyl-benzidine (TMB) (Sigma-Aldrich Co., St. Louis, MO, USA) as the substrate and blank-corrected optical density at 450 nm was measured with a Multiscan ELISA Plate Reader (Thermo Electron Corporation, USA). Serial dilutions of each test serum samples were applied and antigen-specific titers were determined by GraphPad Prism version 5 for Windows (GraphPad Software, La Jolla California USA) using the one site binding hyperbola function of non-linear regression curve fit. IgG titers are given as half-maximal values (inflexion point titer). For measurement of total IgG levels standard curves were constructed by using affinity purified rabbit IgG (Sigma-Aldrich Co., St. Louis, MO, USA) and serum IgG concentrations were determined based on the blank-corrected absorbance values at 450 nm interpolated from the linear portion of the standard curve. Samples were assayed in duplicates for titer and in triplicates for total IgG measurements.

### Statistics

Statistical differences were assessed by pairwise comparisons of relevant groups using permutation tests. Briefly, values from the groups to be compared were randomly reassigned to two groups and the difference between the group means was calculated. Distribution of 5000 randomizations was drawn and the two-tailed *P*-value corresponding to the real sample assignments was determined. The arithmetic mean of 50 such *P*-values was accepted as the probability of α-error. Values of *P*<0.05 were considered significant and were indicated as follows: *, *P*-<0.05; **, *P*-<0.01; ***, *P*-<0.001.
